# An Unusual Case of Deep Vein Thrombosis and Concurrent Necrotizing Fasciitis Following a Fall

**DOI:** 10.7759/cureus.33934

**Published:** 2023-01-18

**Authors:** Muhammad U Rohail, Ayub Khan, Jane Maloof

**Affiliations:** 1 Medical School, West Virginia School of Osteopathic Medicine, Lewisburg, USA; 2 Radiology, Kanawha Valley Radiologists, Charleston, USA

**Keywords:** fall injury, nf, dvt diagnosis, methicillin-sensitive staphylococcus aureus, necrotizing soft tissue infection, necrotizing fasciitis (nf), cellulitis, deep vein thrombosis (dvt)

## Abstract

A 74-year-old male patient presented to the emergency department following a fall with signs and symptoms consistent with right lower extremity (RLE) deep vein thrombosis (DVT) and non-specific skin changes. Further imaging confirmed the initial diagnosis of DVT, and the patient was appropriately treated. However, his condition continued to deteriorate with worsening overlying skin changes, which prompted a computed tomography (CT) scan of his right femur without intravenous (IV) contrast. This revealed fluid tracking along the lateral compartment muscles, which raised suspicion of an abscess. Suspicion for necrotizing fasciitis (NF) was raised with a subsequent CT of the right femur with IV contrast that demonstrated a considerable increase in rim-enhancing fluid collections intramuscularly and extending into both the anterior and posterior compartments, likely correlating with increasing intermuscular abscesses. On imaging, no subcutaneous emphysema or gas accumulation was found, which is a common finding in NF. However, necrotic-appearing muscle was found on surgical debridement and wound cultures confirmed the diagnosis of methicillin-resistant Staphylococcus aureus (MRSA) NF. The patient was then treated with appropriate IV antibiotics and was discharged to long-term inpatient wound care. Similar presentations of DVT and NF made a prompt diagnosis of NF difficult, and it highlights the need for further imaging to rule out NF when a patient has a confirmed diagnosis of DVT.

## Introduction

Necrotizing fasciitis (NF) is a fatal necrotizing soft tissue infection (NSTI) that is rapidly progressing and requires prompt diagnosis for the best prognosis [1,2]. It is typically caused by gram-positive cocci such as Streptococcus pyogenes and Staphylococcus aureus. Risk factors can include diabetes and alcoholism. Signs and symptoms of NF include erythema, swelling, tenderness, and tachycardia, and this presentation is shared with patients presenting with deep vein thrombosis (DVT) [1]. Our patient presented with these symptoms following a fall and was ultimately diagnosed with DVT and concurrent NF.

## Case presentation

A 74-year-old male with a history of chronic kidney disease presented to the emergency department (ED) following a mechanical fall and a two-day history of right lower extremity (RLE) pain and swelling. Per emergency medical services (EMS), the patient was severely hypotensive (60/40), tachycardic, tachypneic, pale, and diaphoretic. No clear cause for the fall was identified. The physical examination revealed generalized tenderness, erythema, swelling of the RLE sparing the right foot, and restricted range of motion in the right hip and knee. Visual inspection revealed two small abrasions to the medial aspect of the right thigh. Doppler ultrasound of his RLE showed an acute DVT of his right popliteal vein. A heparin bolus and drip were administered in the emergency department (ED), and he was subsequently admitted for further management of the DVT and infectious cellulitis of the right leg. Myocardial infarction, stroke, and pulmonary embolism were effectively ruled out through clinical examination, imaging, and lab work.

**Figure 1:**
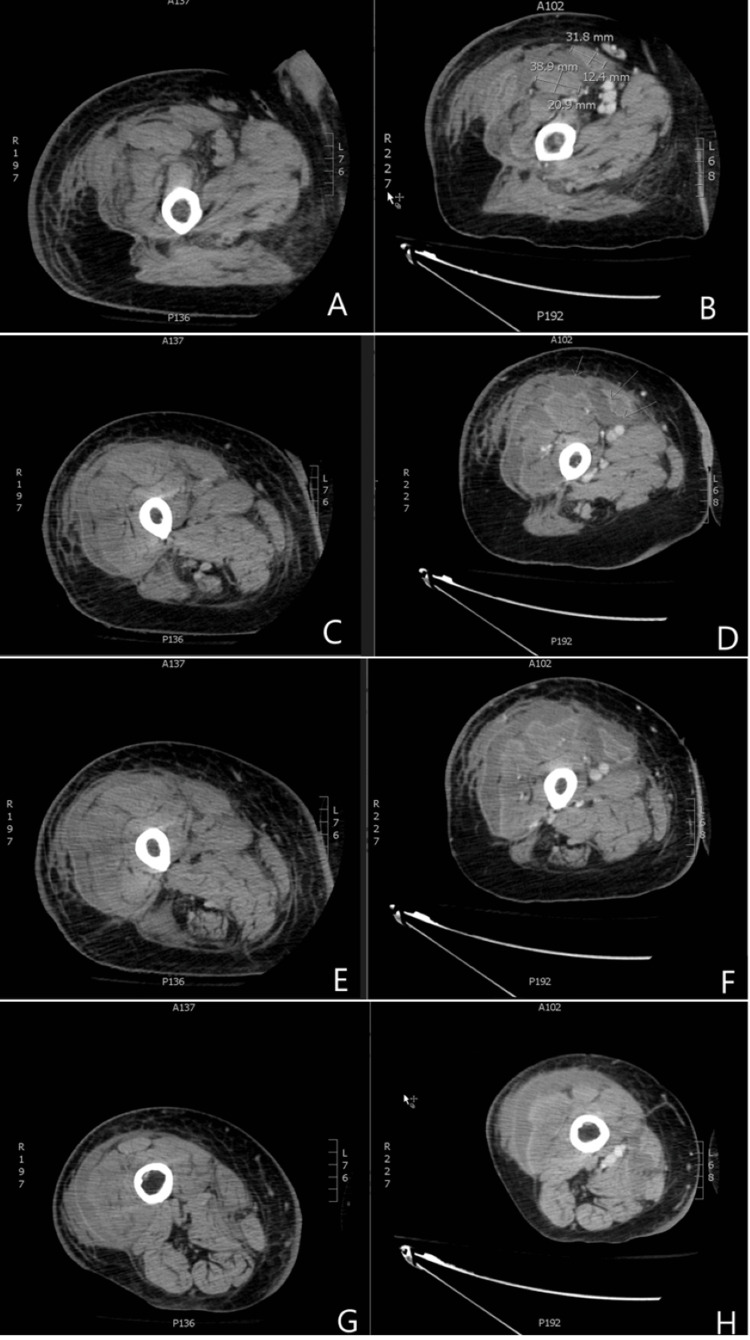
CT of the right femur #1 and #2 CT femur #1 (A, C, E, and G) without IV contrast and CT femur #2 (B, D, F, and H) with IV contrast were performed five days after CT femur #1. Transverse CT images of the right femur show cross sections descending from superior to inferior. The images on the left demonstrate initial findings that were suspicious of a soft tissue skin infection, and the images on the right were taken five days later, demonstrating the development of extensive necrotizing fasciitis. Images B and D show intra- and intermuscular abscesses with a concomitant reduction in muscle volume and size, namely the sartorius, rectus femoris, vastus medialis, vastus intermedius, and vastus lateralis. Images F and H show increasing rim-enhancing lesions of the medial compartment with necrosis of the adductor brevis, adductor longus, and adductor magnus. CT: computed tomography; IV: intravenous

A CT of the right femur without intravenous (IV) contrast (Figures 1A, 1C, 1E, 1G), indicated for worsening cellulitis, revealed fluid tracking along the lateral compartment muscles, which raised suspicion for an abscess. Cultures of the right knee revealed methicillin-resistant Staphylococcus aureus (MRSA), and he was diagnosed with pyomyositis, which was then treated with daptomycin. Subsequent CT of the abdomen and pelvis (CTAP) with IV contrast demonstrated extensive subcutaneous soft tissue swelling and stranding with skin thickening of the right hip joint with fluid within the visualized intramuscular fat planes of the right thigh. However, no rim-enhancing collections were noted. An MRI of the right femur without contrast performed the next day demonstrated RLE edema and fluid in the subcutaneous soft tissues and muscle planes. This was incompletely evaluated due to the patient's inability to tolerate the study secondary to pain. Moderate suprapatellar joint effusion was also noted.

**Figure 2:**
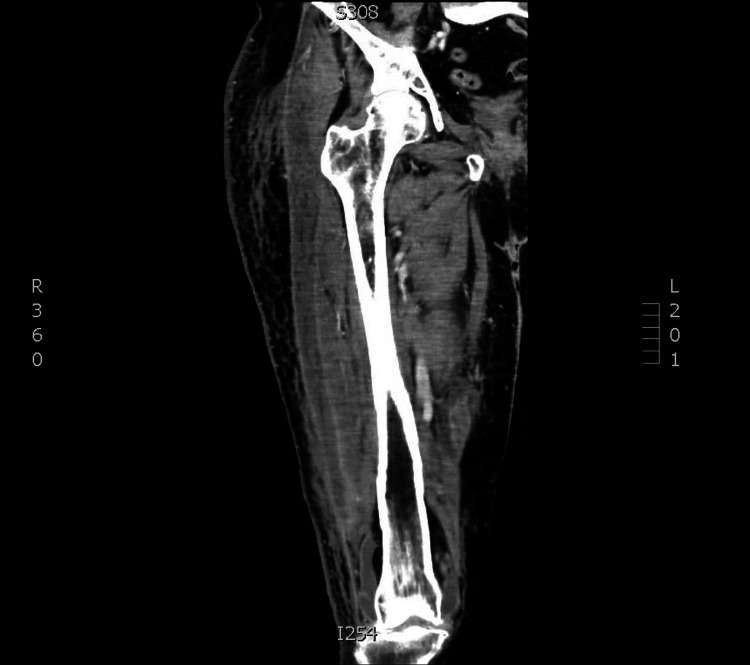
CT of the right femur #2 The rim-enhancing lesion extends the entire iliotibial band in the coronal view CT of the right femur #2 with IV contrast. Subcutaneous stranding indicative of cellulitis is also noticeable on the entire lateral aspect of the right femur.

**Figure 3:**
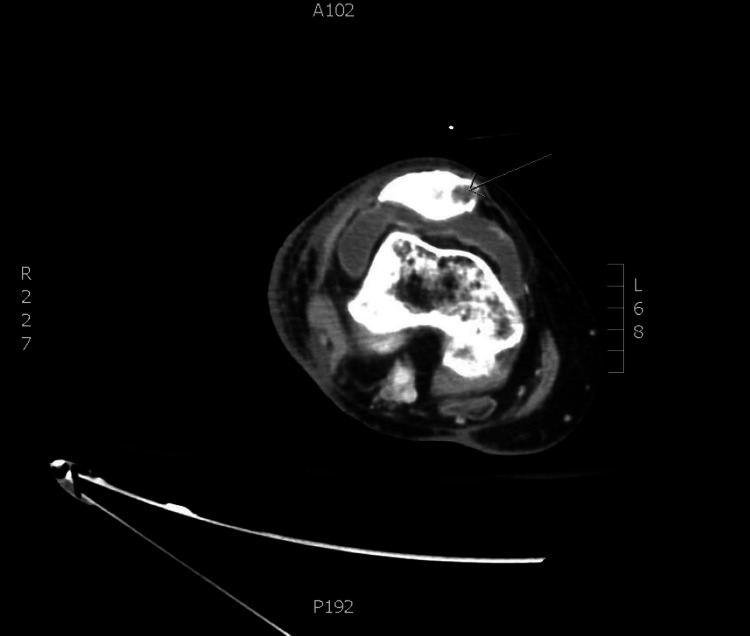
Osteomyelitis of the right knee A large right knee effusion with enhancing synovium and osteolytic nidus medial patella (arrow) is seen in a transverse view of the right knee on CT of the right femur #2 with IV contrast. Septic arthritis and osteomyelitis are confirmed at debridement surgery.

A CT of the right femur with IV contrast (Figures 1B, 1D, 1F, 1H, 2) was performed a few days later and demonstrated a considerable increase in rim-enhancing fluid collections intramuscularly and extending into both the anterior and posterior compartments, likely correlating with increasing intermuscular and intramuscular abscesses. No subcutaneous emphysema was noted. Patellar osteomyelitis was also noted on imaging (Figure 3). These findings raised concern for the presence of necrotizing fasciitis (NF), which was not clearly evident in the clinical examination. Arthrotomy of the right knee was performed with drainage of an abscess. An incision and drainage of the deep abscess were also performed, along with a fasciotomy of the lateral compartment. Fasciitis was noted with a large abscess extending superficially and deeply to the fascia over the vastus lateralis (VL) as well as the iliotibial (IT) band. This extends from the area just proximal to the knee, anterior and lateral, almost to the hip. The VL muscle revealed pus and necrotic-appearing muscle. Cultures of the knee joint revealed MRSA. The patient was continued on daptomycin, and IV clindamycin was started. The patient’s condition continued to improve while in the hospital.

The patient was later discharged to a long-term acute care (LTAC) facility. Subsequent examination by the internal medicine team confirmed the diagnosis of NF, and IV antibiotics and wound care treatment were continued.

## Discussion

NF is a rare and lethal form of NSTI, with a current incidence of 1000 cases per year in the United States [2]. The most common cause of monomicrobial NF is Group A Streptococcus pyogenes (GAS); however, our case presented with MRSA infection, which is an uncommon causative agent [3]. NF rapidly extends superficially and deeply to the fascia, typically after inoculation through a break in the skin or less commonly through hematogenous spread. Progressing across the fascia is easier due to its comparatively lower vascularity than muscle [1,4,5]. It is essential to diagnose NF early since fulminant infection can lead to septic shock, loss of extremities, and eventually death [1,4].

Possible differential diagnoses for a patient presenting with hypotension, tachypnea, and RLE swelling and tenderness include NF, cellulitis, pyomyositis, and DVT. NF is a clinical diagnosis made with supporting evidence from imaging and surgical exploration [4,6]. In our patient, the physical presentation and history of swelling and tenderness guided an initial diagnosis of DVT of the right popliteal vein and concomitant cellulitis of the right lateral thigh. However, despite adequate treatment of the DVT, a follow-up CTAP performed to investigate possible thrombosis of the iliac vein revealed an alarming finding of soft tissue swelling and edema within the intramuscular fat planes of the right thigh. This finding prompted additional imaging of the right femur.

CT imaging is the initial imaging modality of choice for NF and typically reveals fat stranding, fluid collections, and fascial thickening, and these findings are consistent with our CT imaging [4,6]. Classical imaging features of NF include gas accumulation along fascial planes and soft tissue emphysema. Our CT imaging did not demonstrate these findings; however, it is suggested in the literature that the lack of gas accumulation may have been because our imaging was performed at an early stage of infection [6,7]. Visualization of fluid accumulation in both the anterior and posterior compartments of our patient’s leg further demonstrated the multicompartmental fascial spread of the NF. This finding helped shift our diagnosis away from pyomyositis towards NF since it is a distinctive feature of NF not routinely seen in pyomyositis [8]. Although MRI is the imaging modality of choice for soft tissue and may have provided a clearer visualization of the NF, our patient could not tolerate the imaging due to pain, so it was unclear and inconclusive [6,7]. Imaging for NF is often nonspecific and can share similar presentations with other soft tissue infections, so its main value for our patient was localizing the extent of soft tissue involvement, and our findings guided immediate surgical exploration.

Standard NF management includes immediate surgical exploration and debridement of necrotic tissue, as well as concurrent IV antibiotics. In our case, a surgical examination of the right thigh identified necrotic-appearing musculature that corroborated the imaging findings for NF. Additionally, wound cultures are essential in finding the source(s) of infection and guiding antibiotic treatment; however, empiric treatment with broad-spectrum antibiotics can be started prior to the pathology report [2,4,9]. This was consistent with our antibiotic treatment plan. Long-term care for a patient with NF requires a multidisciplinary team for continued monitoring and wound debridement [2].

It is unclear whether the initial pathology was DVT or NF. The patient’s DVT was identified first, and with the aid of CT imaging, NF was subsequently discovered. According to the authors, the patient's right medial thigh abrasions from his fall served as the site of inoculation for MRSA infection, which later progressed to NF.

A case of an adolescent male presenting with DVT, fibular osteomyelitis, and NF has been reported with a concomitant infection of community-acquired MRSA (CA-MRSA) that had similar nonspecific findings on imaging [10]. This was the only other case we found that had DVT, osteomyelitis, and NF, all potentially caused by a CA-MRSA infection. It was demonstrated in a study that looked at patients with lower leg erysipelas that 10% of the patients had a DVT of the same leg [11]. It is also possible for DVT to mimic the early clinical findings, such as erythema, swelling, and tenderness, of NF, as was the case with an obstetric patient who was initially treated for a thromboembolic disease but in actuality had NF [5]. Our case demonstrated a unique incident of DVT and NF occurring simultaneously, and prompt diagnosis of the necrotizing soft-tissue infections (NSTI) with imaging was crucial in reducing potential mortality in this patient.

## Conclusions

Our case represents an unusual incidence of a DVT with a concurrent underlying NSTI. The presenting signs and symptoms of DVT are shared by many different etiologies, such as soft tissue infections. Therefore, it is imperative for clinicians to keep lethal conditions, such as NF, on the ongoing list of differentials when treating the initial DVT. The authors believe that with an initial diagnosis of DVT, prompt investigation with further imaging should be considered. This may aid in the rapid diagnosis of additional fatal pathologies, such as NF, that may be masked by a DVT.
